# Experimental Lab Tests on Rabbits for the Optimization and Redesign of Low-Cost Equipment for Automated Peritoneal Dialysis

**DOI:** 10.3390/bioengineering11020114

**Published:** 2024-01-24

**Authors:** Sergio Rodrigo Méndez-García, Edgar Cano-Europa, José Ocotitla-Hernández, Margarita Franco-Colín, Oscar Iván Florencio-Santiago, Christopher René Torres-SanMiguel

**Affiliations:** 1 Instituto Politécnico Nacional, Escuela Superior de Ingeniería Mecánica y Eléctrica, Nacional Unidad Zacatenco, Sección de Estudios de Posgrado e Investigación, Ciudad de México 07738, Mexico; 2 Instituto Politécnico Nacional, Escuela Nacional de Ciencias Biológicas Unidad Zacatenco, Ciudad de México 07738, Mexicooflorencio1400@gmail.com (O.I.F.-S.); 3 Instituto de Seguridad y Servicios Sociales de los Trabajadores del Estado, Dirección de alta Especialidad, Ciudad de México 14070, Mexico

**Keywords:** biomechanics, nephrology, dialysis, experimental lab test, nephrectomy, rabbit

## Abstract

This work shows the experiences acquired by the experimental test performed to validate an automated peritoneal dialysis machine using rabbits with kidney damage to find improvements that can be made for future advances. These are listed to understand the direction of the development of the machine. The article shows the device’s background and previous tests using a testbed. The rabbit anatomy was prepared for nephrectomy surgery. The tests were practiced by checking all of the APD machine’s subsystems. The data were analyzed to develop improvements in the process. The results indicate the importance of the DPA machine as an alternative by implementing peristaltic pumps to substitute disposable cassettes. The identified improvements are the main objectives for research to continue improving the technology.

## 1. Introduction

Experiments on animals are essential for global technological advances, medical devices, and discoveries. Historical events have revealed the importance of animal validation before being applied to humans. Pharmacological elements are discovered to prevent or eradicate diseases; a significant difference from the past is that animal studies have increased the effectiveness of those medicines. This is because they have undergone all of the necessary improvements to ensure they do not harm the human body, even detecting toxins or elements harmful to humans [1]. Moreover, the advances in technology and biomaterials that can be used to repair or substitute elements in humans best demonstrate the importance of animal experimentation. This is not only useful for this purpose, but can also help to cure or prevent issues in animals. These biomaterials can be used to recreate body parts and insert them where needed [2].

There are protocols in place for animal experimentation to ensure safe practice for animals [3]. Digital simulations have been developed to be as similar to real-life scenarios as possible to reduce the likelihood of issues with experiments [4]. Following simulations and in vitro tests, some devices require practical testing to confirm that there will not be any damage to, or developments of any bacteria in, an actual patient [5]. Animal testing is even used to make successful advances and improvements to surgical procedures [6,7]. The animals are constantly evaluated to ensure they have the best conditions to live in before and after experimentation [8]. Additionally, techniques to be employed in dangerous situations involving animals have been established [9]. Because animals develop diseases like humans, research can point to a cure or correct treatment [10,11,12,13]. Technological advances, even in the future, will not require animal validation, or at least they will be less invasive than in different procedures. Urine collection to determine pathogens, for example, does not harm animals when studying their organs [14,15,16]. Chronic kidney disease (CKD) is an important topic nowadays, because overweight and hypertension are increasing in the population due to poor dietary habits and a lack of exercise [17].

In México, the incidence CKD is reaching dangerous levels, as evidenced by the number of cases of diabetes diagnosed in the country. The therapies used for this disease are expensive, so public institutes provide machines for patients to self-practice the complete treatment. Still, this is insufficient for the population [18,19]. The first research in this field was documented in the 18th century, involving the first test on peritoneal damage and the treatment of Christopher Warrick [20]. Since then, technological advances have helped to create devices that allow relevant therapies to be practiced [21]. Over time, improvements in these processes have become more significant, but nowadays, the most well known machine [22] has many factors that need to be improved. This is the purpose of this research centered around an automated peritoneal dialysis (APD) machine that uses peristaltic pumps instead of disposable cassettes. Its program allows us to personalize the therapy for each patient [23].

This APD machine was developed as a tangible device to help reduce the risks of CKD. However, it must be tested for validation purposes at safe institutions responsible for not risking people’s health [24]. This begins with in vitro tests that evaluate the machine and ensure it does not reach dangerous levels for users, necessitating a testbed for validation. However, various variables must be considered to obtain the best results [25].

The testbed was also developed to evaluate scenarios for different patients in therapies similar to real ones, while sensors read the variables used during treatment [26]. As a reference, the physical methods used to evaluate devices are not the most advanced, but they are necessary for experimental results. Additionally, even virtual tests can be conducted [27]. Another advancement used is connectors for peritoneal dialysis to substitute in case of any error with the dialysate bag [28]. There are different methods that can be used to check if the machine is working correctly, but more than the machine itself, the therapy in general is not a meaningful way of validating the device [29,30].

An experimental test was required to validate the APD machine, and it can be improved to yield better results and confirm that it does not harm patients who use it. Chronic kidney diseases can be treated in animals, with factors such as age playing a role. It is also hereditary in animals. The machine will help to reduce the amount of CKD cases.

## 2. Materials and Methods

**Figure 1:**
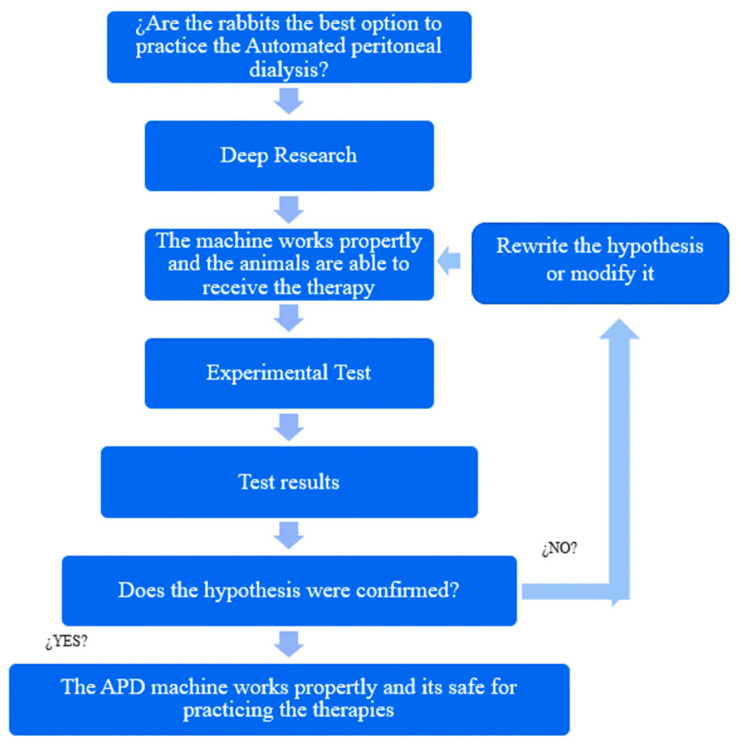
Experimental methodology.

The methodology used for this research was experimental and is described below in Figure 1.

### 2.1. Automated Peritoneal Dialysis (APD) Therapy

**Figure 2:**
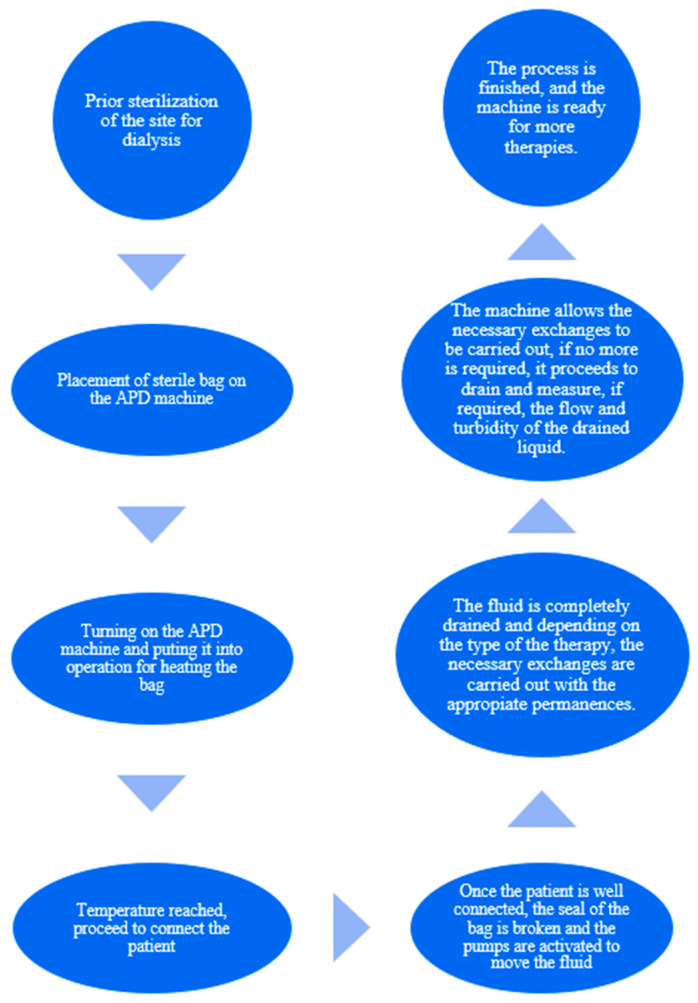
Description of the automated dialysis process.

The main process for the APD machine is described below in Figure 2.

### 2.2. Automated Peritoneal Dialysis (APD) Machine

**Figure 3:**
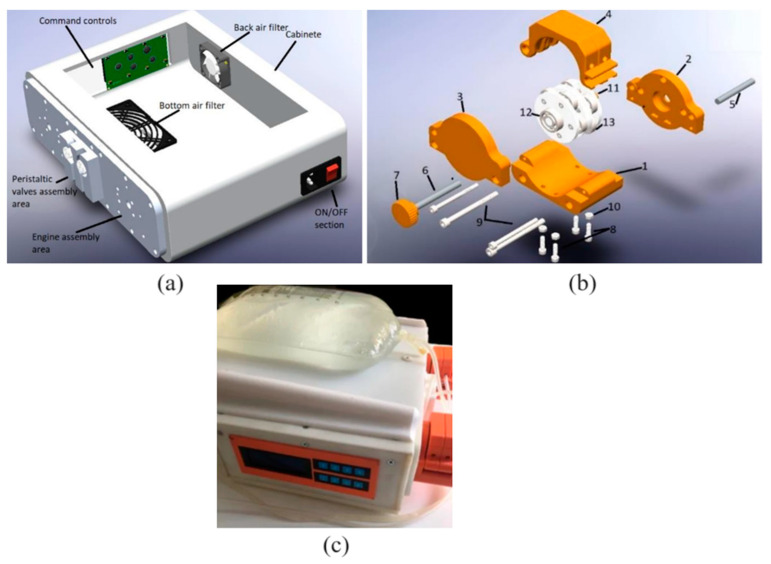
(**a**) APDM design, (**b**) Peristaltic pump exploded view, (**c**) APD machine with dialysate fluid bag. Reprinted from Ref. [23].

An automated peritoneal dialysis (APD) machine is a device used to practice therapy in an automated way. This is recommended to increase CKD patients’ life expectancy and not invade their everyday activities. The APD machine (APDM) showed in Figure 3, was developed to improve the standard method using disposable cassettes that allow the fluid of the dialysate fluid bags to be transported. This is substituted by peristaltic pumps which transport the fluid through the roller mechanism outside the hoses and continue pressing them to control the quantity and velocity of the liquid. This is a general innovation of the machine. The functioning of the APDM is typical, involving three processes: the mechanic subsystem, the electronic subsystem, and the control subsystem. The mechanic subsystem contains the heating process, hose calculation, and the partial design of pumps. The electronic subsystem comprises the selection and design of elements that make the system work. Finally, for the control, the subsystem stores the algorithm that controls all of the components [23].

### 2.3. Testbed for Validation of the APD Machine

**Figure 4:**
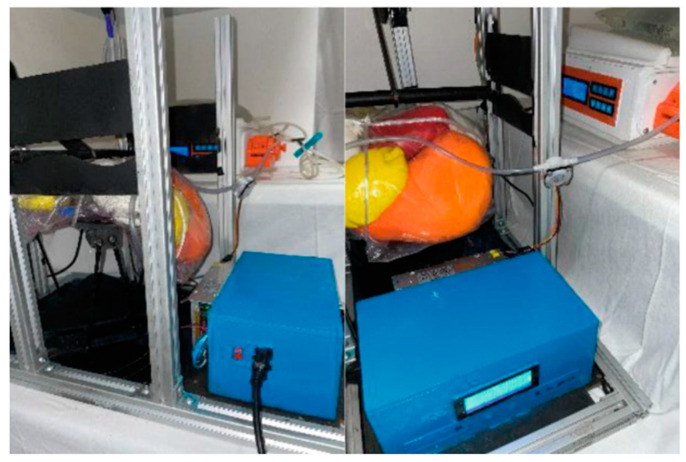
The testbed is connected to the APDM. Reprinted from [26].

After the APDM was designed and built, it was partially improved during its development using many in vitro tests (Figure 4). A testbed was designed specifically for testing this device to the experiment without adding pressure for the first time and to check if the sensors added to the testbed were read correctly. Tests were conducted with three different situations, adding other pressures and temperatures [26]. The APDM successfully passed the tests and continued the steps to confirm that the machine was not harmful to animals. The following figure shows the testbed connected to the APDM, simulating a therapy of automated peritoneal dialysis.

### 2.4. Development of Chronic Kidney Disease (CKD) in Rabbits as a Model of Peritoneal Dialysis

CKD was induced by the heuristic model 5/6 nephrectomy (5/6 NFx). Five male rabbits weighing 2.92 ± 0.25 kg were maintained in individual cages at 21 ± 2 °C, 40–60% relative humidity, in a 12:12 h light/dark cycle with lights on at 8 AM. Food (ConejinaN Purina™, Vervey, Suiza) and purified water were provided *ad libitum.* The experimental procedures were carried out by the provisions of the [NOM-062-ZOO-1999] Norma Oficial Mexicana, Mexico [31], World Organization for Animal Health and the Association of American Veterinary Medical Colleges.

**Figure 5:**
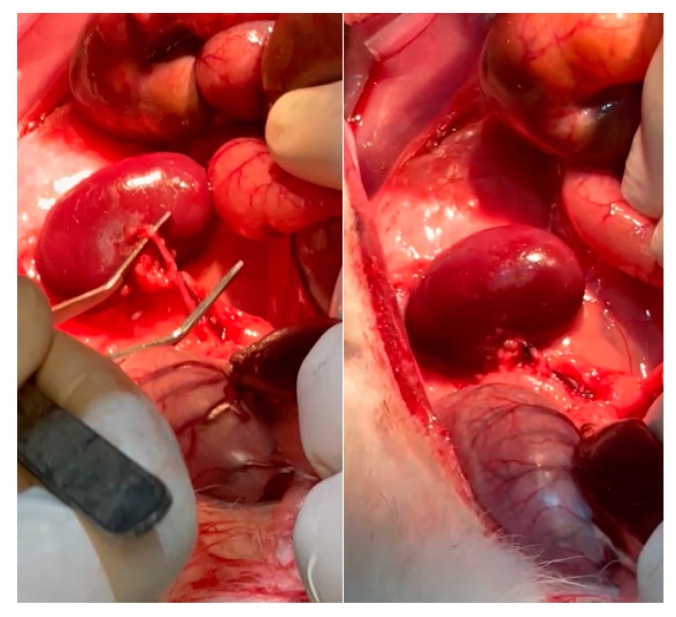
Nephrectomy. Kidney vessels were located and occluded (**left**). Infarction 2/3 of the kidney was induced (**right**).

Animals were anesthetized with ketamine (25 mg/kg) delivered intramuscularly (im) and pentobarbital–xylazine delivered intraperitoneally (ip) (20 and 2 mg/kg, respectively). Then, ventral laparotomy was performed under aseptic conditions, and two of the three left renal blood vessel branches were ligated with 2-0 black silk (Figure 5). Afterward, the contralateral kidney was nephrectomized. After the surgery, tramadol (10 mg/kg) by oral gavage (og) and enrofloxacin (50 mg/100 mL) in purified water were administered for three days to avoid pain and prevent infections. After four weeks, renal function tests were performed.

### 2.5. Catheter Implantation

**Figure 6:**
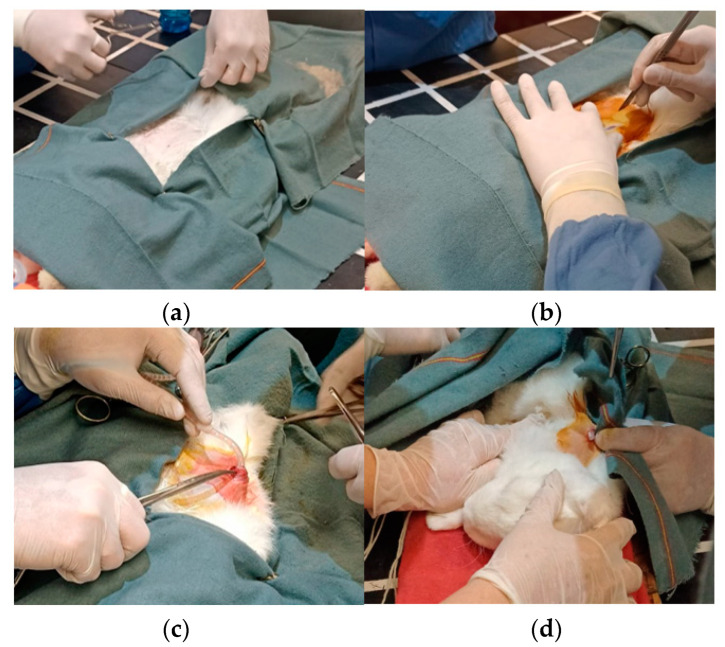
Catheter placement surgery. (**a**) The area preparation; (**b**) the first contact with the peritoneal area; (**c**) the catheter placement; and (**d**) the relocation of the manipulated catheter to a better area where the patient could not hurt himself.

Four weeks after surgery, the animals meeting the following serum criteria (mg/dL) were found to be suitable for peritoneal catheter implantation: blood ureic nitrogen (BUN) ≥ 20, uric acid ≥ 5, creatinine ≥ 6, creatinine clearance ≤ 2 (mL/min), hematocrit ≤ 40%, proteinuria, and hematuria. The animals were anesthetized with ketamine (25 mg/kg, im) and pentobarbital–xylazine (20 and 2 mg/kg, respectively, im). In deep anesthesia, a peritoneal catheter (peritoneal dialysis catheter; Curl Cath, 2 Cuff 57 cm; Argyle™, CA, USA) was introduced into the peritoneal cavity. The catheter was tunneled through the back and exteriorized, fixed with 0 black silk suture to prevent it from being damaged, and connected to the APD machine, as it’s shown in Figure 6. After surgery, tramadol (10 mg/kg, og) and enrofloxacin (50 mg/100 mL) in purified water were administered to avoid pain and prevent infections. 

### 2.6. Automated Peritoneal Dialysis (APD)

Before the APD, three quick washes with 10 mL of peritoneal dialysis fluid (lactate-buffered 1.5% glucose-containing fluid; (Baxter Healthcare, IL, USA) were performed to clean the peritoneal cavity. Then, 30 mL/kg of peritoneal dialysis fluid was infused for one hour using the APD machine. 

### 2.7. Uranalysis and Renal Function Evaluation

The day before surgery and four weeks later, animals were placed in individual cages to collect 24-h urine. Urine was analyzed using reagent strips (Mission^®^, ACON, CA, USA), and the sediment was examined by microscopy. While urine samples, heparinized blood was obtained from the vena *auricularis laterialis*. Microhematocrit was evaluated, and afterwards, the serum was separated. Proteinuria, azoated (BUN, creatinine, and uric acid), lactate, and electrolytes (sodium and potassium) were measured using Randox kits (Randox, WV, USA). 

### 2.8. Statistical Analysis 

All data are reported as the mean ± standard error of the mean (SEM). Basal and four-week 5/6 NFx body weight, renal function, electrolytes, and hematocrit were examined by paired *t*-tests. The monitoring of azoated and electrolytes during APD was analyzed by repeated measures (RM) and one-way analysis of variance (ANOVA). The ANOVA was followed by the Student–Newman–Keuls (SNK) *post hoc* test. Statistical significance was considered at *p* < 0.05.

## 3. Results

Table 1 shows that 5/6 NFx induces CKD and causes increased BUN, uric acid with proteinuria, and hematuria. In addition, CKD decreases body weight, creatinine clearances, and microhematocrit.

**Figure 7:**
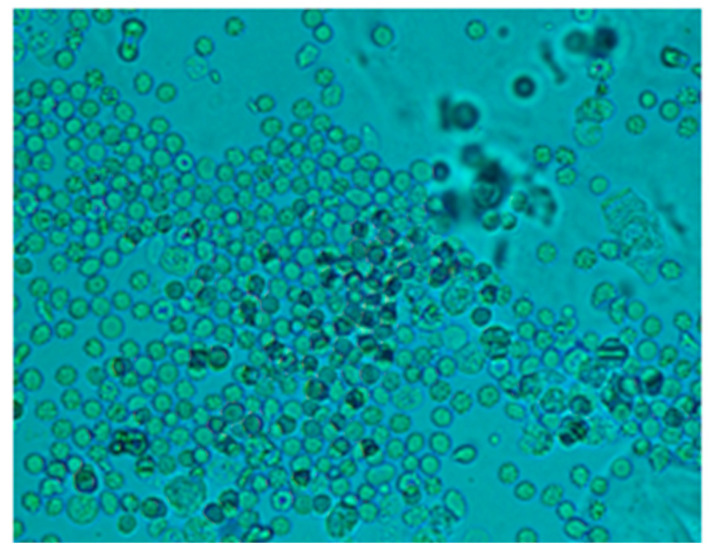
Representative light micrograph of urine sediment from rabbits four weeks after 5/6 NFx at 40×. The presence of multiple haematids is a sign of CKD development.

Moreover, abundant haematids in the urine sediment of CKD rabbits are shown in Figure 7.

**Figure 8:**
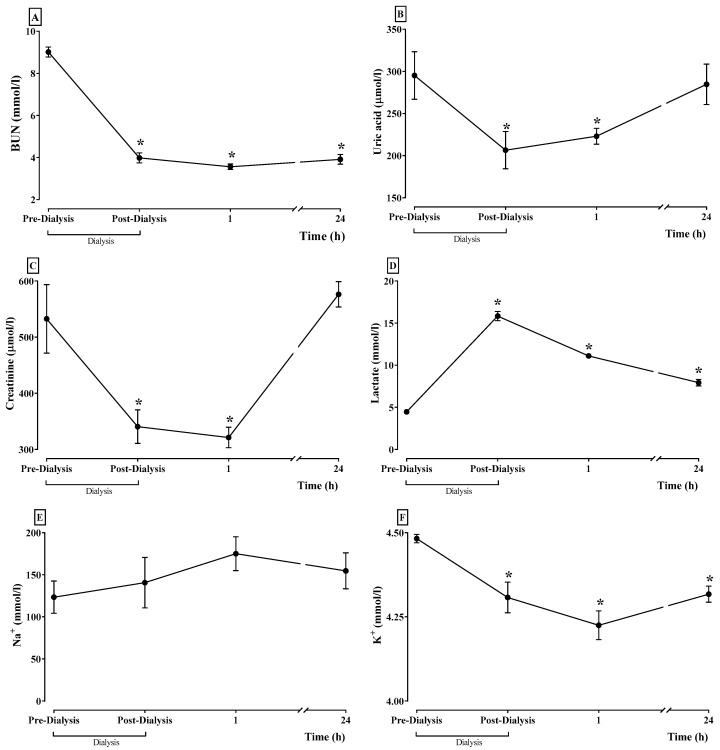
The BUN concentration (**A**), uric acid (**B**), creatinine (**C**), lactate (**D**), sodium (Na^+^) (**E**), and potassium (K^+^) (**F**) of rabbits with CKD after PD. Data represent mean ± SEM. (*) *p* < 0.05 compared to the Basal. RM one-way ANOVA and SNK *post hoc* test.

Figure 8 illustrates the levels of BUN (A), uric acid (B), creatinine (C), lactate (D), sodium (E), and potassium (F) before and after APD. All these biochemical markers are elevated before the APD. Meanwhile, the APD reduced them except for sodium and lactate. The last one increased after APD. 

The therapy was calculated depending on the rabbit’s weight. This is to infuse the correct quantity of dialysate fluid, not to damage the patient. The permanence is equal for all the tests, one hour with the fluid inside the peritoneum. Finally, the drain is made to extract all the collected dirty fluid and to check if the rabbit is not retaining some fluid inside because of filtrations or something else.

As the Table 2 shows, the rabbits had some troubles during the draining time because the fluid was not the exact amount; this is probably because they could retain or absorb the liquid during the permanence time, but it is essential to remark that it was in the first permanence. As a positive result for the APD machine, the infusion times were correct according to the volume drained, so the peristaltic pumps appropriately worked. Some adjustments to the power have to be improved, and for the universal connections, there is a method to be practiced in future tests.

During the therapies, many factors were found to improve the function of the APD machine, shown in Table 3. Therefore, it will be implemented to get a better result and the machine’s functionality.

The machine fulfilled its function, i.e., heating the dialysate bag, allowing it to connect the hoses to the patient and turn on the peristaltic pumps. The fluid was able to pass to the patient properly, and the infusion time depended on the volume infused. However, with some of the extraction methods using measured syringes, it was possible to check if the volume infused was the same as the volume drained. There was no presence of peritonitis issues. The patients with the first infusions showed a significant improvement in their values. For the final time, when the patients were no longer sedated, they usually moved, and the catheter was not damaged because of the location.

Table 1 shows 5/6 NFx-induced CKD after four weeks, and all the animals were suitable for dialysis catheter implantation. In addition, the APD machine was able to infuse and drain all the dialysis fluid. 

**Figure 9:**
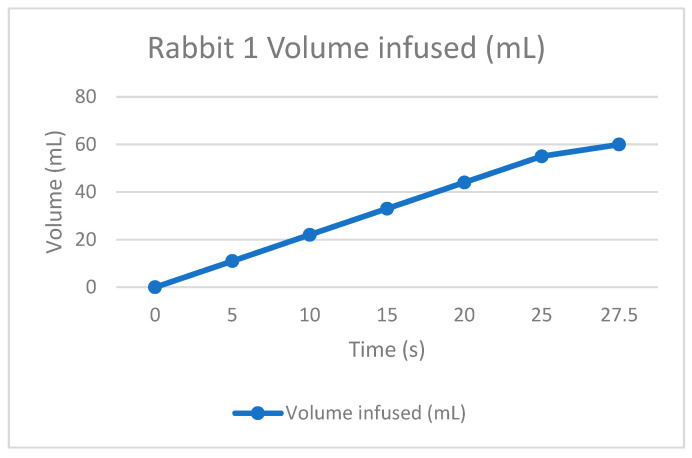
Volume infused against the time of rabbit number 1.

**Figure 10:**
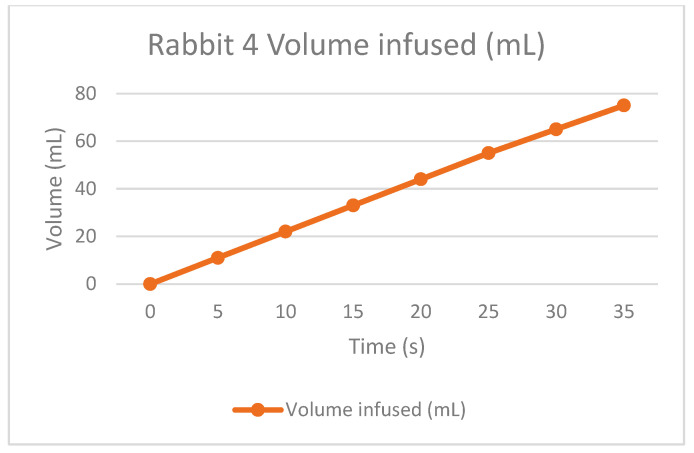
Volume infused against the time of rabbit number 4.

**Figure 11:**
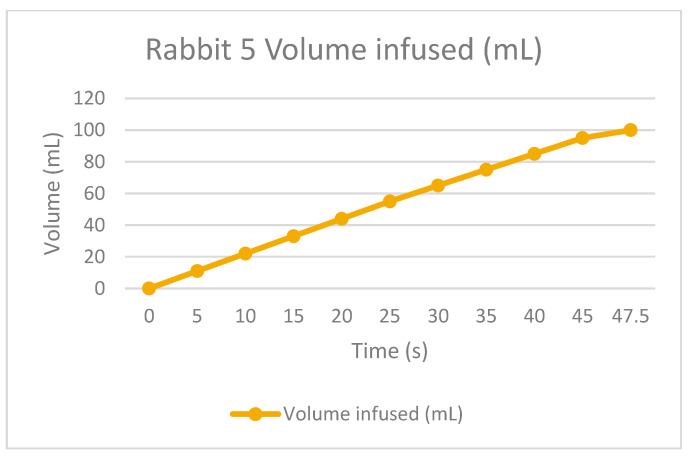
Volume infused against the time of rabbit number 5.

**Figure 12:**
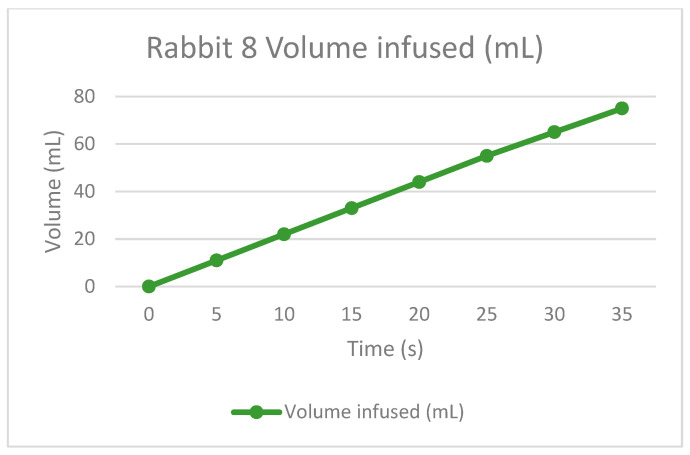
Volume infused against the time of rabbit number 8.

The following graphs of Figure 9, Figure 10, Figure 11 and Figure 12 show the volume-infused behavior, representing the constant flow that the peristaltic pumps allowed for transporting during each therapy, according to the time-based results.

As an important note, the first rabbit underwent three therapies, but the final session resulted in dehydration of the patient; therefore, for the rest of the rabbits, the number of treatments was reduced to two per day. This is because the levels mitigated the risks with the second therapy, and the patients were better.

Thanks to the analysis of the machine during the tests, many ways of optimizing the device were identified. The main objective of conducting these tests was to understand if the machine works correctly or if any issues exist that need correcting for the future of the technology.

The corrections are listed below. The first two improvements are related to the use of the equipment, while the last are focused on the redesign of the machine.

1. The machine should indicate the optimal height of the work area, which should be elevated next to the patient to minimize pressure leakage from the dialysis bag in the last fluid volumes.
2. The peristaltic pumps should be checked to assess the condition of the motor and ensure its functionality during therapy.
3. Adding a turbidity sensor should increase the technological level of the machine, and an alarm system should inform the patient if something goes wrong during or after the therapy.
4. The sterility of the machine should be increased by changing the materials used in manufacturing to reduce the contamination zones.
5. For the final prototype, the machine should be programmed to operate with minimal human intervention.

**Figure 13:**
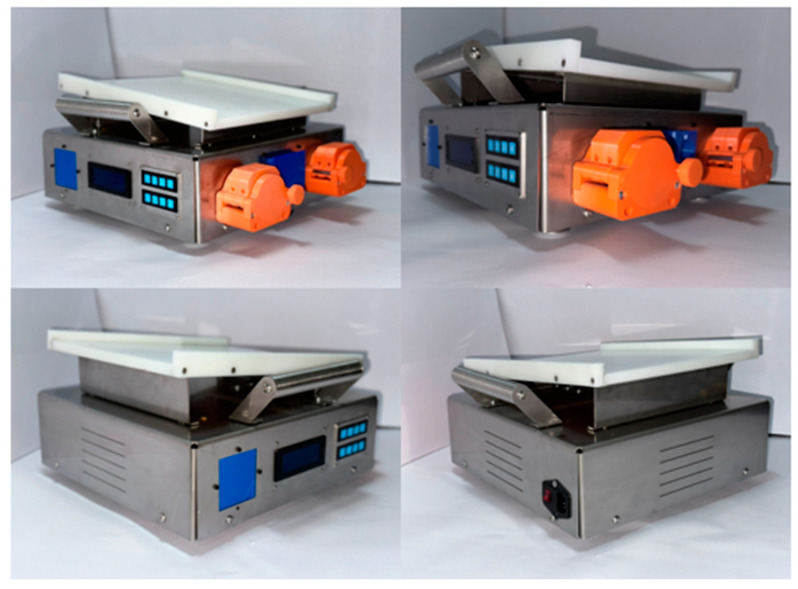
Redesign of the low-cost equipment for peritoneal dialysis.

For the record, improvements in the machine are being developed, as Figure 13 shows below.

The first feature to be improved will be the heat bed for increased heat transfer to the bag. It would be better to use a single element instead of two parts (1). Then, the positioning of the components attached to the structure will be made permanent for patient safety purposes (2). For this design, heat distribution in the heat bed will be increased, but the elements must be protected from overheating. The final design of the vents will be improved to avoid making the structure weak due to cuts (3). The turbidity sensor must be added in a way that does not damage the rest of the equipment and should be isolated from the circuits (4). Finally, the lower cover will be secured to prevent the patients from opening it (5).

## 4. Conclusions

The APD machine facilitated therapy, although there were some drawbacks with the connection to the catheter. The process worked effectively, helping to reduce the BUN levels and allowing the rabbits to even move as if they were healthy. Despite their successful recovery, the machine needs improvements.

Regarding the manipulation of the machine, it is clear what the process needs. As mentioned, the connections need a universal design for easier patient connection to the APD machine. While the weight is manageable, improvements in the transportability are necessary for better handling.

The experimental test concluded that the infusion functioned normally, despite complications during therapy due to connections from the machine to the catheter. Improvements can be made by programming the code with different times for automated processes, rather than relying on measuring the time with an accurate period. This could be substituted with internal commands of the circuit, and the “plug and connect” feature should be added in the next step of the machine’s development to simplify use for the users.

## Figures and Tables

**Table 1:** Clinical parameters of induced CKD.

Variable	Basal	5/6 NFx
Body weight (kg)	3.2 ± 0.1	2.3 ± 0.3 *
Microhematocrit (%)	62.5 ± 2.8	38.8 ± 1.7 *
BUN (mmol/L)	1.61 ± 0.18	25 ± 1.2 8.93 ± 0.43 *
Uric acid (μmol/L)	107.06 ± 11.9	29.7± 29.74 *
Creatinine clearance (mL/min)	1.3 ± 0.3	1.4 ± 0.3 *
Proteinuria (g/24 h)	0.006 ± 0.0006	1.070 ± 0.1158*
Urine volume (mL)	62.0 ± 3.1	38.8 ± 4.3 *

Data represent mean ± SEM. (*) *p* < 0.05 compared to the Basal. Paired *t*-test.

**Table 2:** Therapies on each rabbit results.

Rabbit Number	Number of Quick Washes	Number of Dialyzes	Fluid Infused			Permanence Time	Fluid Drained		
1	3	3	60 mL (2.5%)	60 mL (2.5%)	60 mL (2.5%)	1 h between each infusion	30 mL	60 mL	60 mL
4	3	3	75 mL (1.5%)	75 mL (1.5%)	75 mL (1.5%)	1 h between each infusion	75 mL	75 mL	75 mL
5	3	2	100 mL (1.5%)	100 mL (1.5%)		1 h between each infusion	90 mL	100 mL	
8	3	2	75 mL (1.5%)	75 mL (1.5%)		1 h between each infusion	70 mL	75 mL	

**Table 3:** List of subsystems of the APD machine.

Subsystem	Description
Mechanic subsystem	Peristaltic pumps are composed of five bearings that allow the pressing of hoses and the transportation of a certain amount of fluid per revolution. At the end of the therapy, they transport approximately 2 L in 15 min. The pumps worked adequately during the tests, but the maintenance must be improved.
	For the connection hoses, there were leaks during the tests. Those are designed to eliminate user problems, but the easier connection between the machine, the bags, and the catheter must be improved.
	The system that we used to lock the hoses is well prepared, making it easy to manipulate, and it does not require specific maintenance. It is composed of a safety bolt and hose holder housing.
	The heating base is the one that increases the temperature of the bags, and it is made of a material that conducts the heat from the electronic heating system. Therefore, the bag reaches the temperature depending on the initial values, but the material performs appropriately.
	In general, the structure of the APDM is adequate for easy transport; it is not too heavy, and the dimensions are less than the commercial devices, so it does not take up much space to place it anywhere. However, it can be improved with a handle.
Electronic subsystem	The ignition system is the first place to have contact with the user, so it must be easy to manipulate. The wire connection and the on/off button are good places not to be hard to find. It is even intuitive to identify.
	The heating system, as mentioned, appears in the three subsystems, and it is the principal characteristic of the APDM, which is why the ignition increased the temperature of the bags. This works properly according to the test results.
	The motors of the pumps are the main reason they could have problems in their function, so this requires maintenance to work correctly.
	The motor valve presented the same situation as the motor pumps. Its function was correct during all the tests.
	The wire connections are fundamental for the APDM functionality; they are required to communicate all the electronic elements. If a short circuit must be repaired as soon as possible, this happens before the first therapy, so it is recommended to modify them.
Control subsystem	The CPU controller is responsible for controlling all the mechanisms of the electronic devices and ensuring that the machine will work properly and fulfill the sequencing process. The CPU worked adequately during the therapies except when the wire connections failed once.
	The menu and display are the interfaces that interact with the user directly to show them the therapy state. If they are required to repeat the process, it will work properly, but because of the CPU, it is conditioned for the wire connection, and if it fails, the menu and display will not work.
	In general, programming functions work adequately, the adjustments made for the specific therapy in rabbits are correct, and they can be improved as part of the optimizations of the APDM process.
	The sensors are responsible for reading the values depending on the variable. They provide feedback to the controller so that they can make decisions about the process and display the state of the therapy. Although the temperature sensor worked adequately during the tests, the flow and turbidity sensors were not used to maintain the sterility of the treatment.
	The heating control is part of the APDM, which manipulates the elements responsible for the heating base function. It operates automatically and is adjusted to allow the elements to increase the temperature at a certain level to heat the bag. It worked adequately during the tests.

## Data Availability

Data are contained within the article.
